# Exploratory transcriptomic analysis of mouse articular cartilage in response to tissue inhibitor of metalloproteinase 3 identifies inflammation-associated gene expression changes

**DOI:** 10.3389/fimmu.2026.1794078

**Published:** 2026-03-11

**Authors:** Manuela Mengozzi, Ben Towler, Jordan Kwabiah, Fawad Razmandeh, Fabio Simoes, Lisa Mullen

**Affiliations:** 1 Clinical and Experimental Medicine, Brighton and Sussex Medical School, University of Sussex, Brighton, United Kingdom; 2 School of Life Sciences, University of Sussex, Brighton, United Kingdom

**Keywords:** cartilage, hypoxia, interleukin-17, lipocalin-2, osteoarthritis, serum amyloid A proteins, TIMP-3, transcriptomics

## Abstract

**Introduction:**

Tissue inhibitor of metalloproteinase 3 (TIMP-3) is a broad-spectrum inhibitor of matrix metalloproteinases (MMPs) and ADAM/ADAMTS (a disintegrin and metalloproteinase with thrombospondin motifs) family enzymes that regulate extracellular matrix (ECM) homeostasis. Because these enzymes play key roles in articular cartilage turnover, TIMP-3–mediated inhibition protects against cartilage degradation, a hallmark of osteoarthritis (OA), and has been explored as a therapeutic target. Nonetheless, unexpected detrimental effects of TIMP-3 on bone mass and structure have been reported in transgenic mice overexpressing TIMP-3 in cartilage. Mechanistically, TIMP-3 binds catabolic enzymes and blocks their active sites but also interacts with low-density lipoprotein receptor-related protein 1 (LRP-1) and sulfated proteoglycans in the ECM, processes that regulate its half-life through a balance between endocytosis and ECM retention and may influence cell signaling.

**Methods:**

We investigated whether TIMP-3 affects gene expression in *ex vivo* mouse articular cartilage explants under normoxia or physiological hypoxia (3% O_2_). Femoral head cartilage explants were treated with recombinant TIMP-3 and then processed for RNA sequencing (RNA-seq).

**Results:**

Hypoxia alone induced a strong transcriptional response, confirming the model’s responsiveness, whereas TIMP-3 altered the expression of only a small subset of genes. RT-qPCR validation confirmed TIMP-3–mediated upregulation of inflammation-associated genes, including *Saa3* under both oxygen conditions, and IL-17 signaling pathway genes (*Il17b*, *Mmp3* and *Lcn2*) under normoxia, and downregulation of proliferative genes *Pbk/Topk* and *Racgap1* under hypoxia. Hypoxia alone downregulated all these genes.

**Discussion:**

The distinct transcriptional effects observed under normoxia and hypoxia highlight the importance of accounting for oxygen tension in cartilage studies. Potential inflammation-associated gene expression responses to TIMP-3 should be considered in its therapeutic development for arthritic disease and may inform optimization of treatment strategies.

## 1 Introduction

Extracellular matrix (ECM) homeostasis is essential for maintaining tissue structure and function and is regulated by the interplay between matrix-degrading enzymes and their endogenous inhibitors. Regulation of this balance drives ECM remodeling that occurs during development and continues in adult tissues in both physiological and pathological contexts, including wound healing, inflammation and tumor invasion, and its dysregulation contributes to diseases such as arthritis and cancer (1, 2).

Matrix metalloproteinases (MMPs) are a large family of zinc-dependent endopeptidases that degrade key ECM components such as collagen and proteoglycans and thus play a central role in ECM turnover (1). Their activity is regulated in part by tissue inhibitors of MMPs (TIMPs), a family of four proteins (TIMP-1, -2, -3, -4) that bind MMPs in a 1:1 molar ratio and inhibit their proteolytic activity by blocking the active site via their N-terminal domain (3, 4). Among the TIMP family, TIMP-3 has the broadest inhibitory spectrum. In addition to MMPs, TIMP-3 also inhibits members of the related ADAM family (a disintegrin and metalloproteinase), including ADAM17, also known as TACE (tumor necrosis factor-α converting enzyme), and ADAMTS enzymes (ADAMs with thrombospondin motifs) (5–7). Like other TIMPs, but with higher affinity, TIMP-3 binds to low-density lipoprotein receptor-related protein 1 (LRP-1), a process that promotes the endocytosis of TIMP-3 and its bound MMPs (8, 9). TIMP-3 is unique in its strong binding affinity to ECM components, a property that prolongs its extracellular half-life compared to other TIMPs and enhances its local inhibitory function (10, 11). In addition, it can bind vascular endothelial growth factor receptor 2 (VEGFR2), thereby inhibiting VEGF-mediated angiogenesis (12).

TIMP-3 plays a crucial protective role in articular cartilage (10), where it inhibits several MMPs, including MMP-13, the principal collagenase targeting type II collagen, as well as ADAMTS-4 and -5, also known as aggrecanase-1 and -2 respectively, which specifically cleave aggrecan, the major proteoglycan in the cartilage ECM (6, 9). This inhibitory capacity highlights TIMP-3’s potential as a therapeutic agent in degenerative joint diseases such as osteoarthritis (OA), where excessive ECM degradation in articular cartilage contributes significantly to disease progression (13, 14). Notably, TIMP-3 knockout mice exhibit increased MMP and aggrecanase activities and greater cartilage degradation (15). While cartilage-specific overexpression of TIMP-3 in mouse models of OA was chondroprotective, it also had detrimental effects on bone mass and structure (16, 17).

Mechanistically, TIMP-3 is a well characterized direct inhibitor of protease activity acting at the protein level, as extensively demonstrated in many *in vitro* studies (3, 4, 6, 9). Whether TIMP-3 has broader biological effects, such as regulation of gene expression, has not been a primary focus of previous studies, although one report has described transcriptional effects in cardiomyocytes (18). To address this, we examined whether exposure to TIMP-3 alters gene expression in articular cartilage isolated from the femoral heads of mice and used as an *ex vivo* explant model, as previously described (19, 20). Although only a small fraction of cartilage tissue is cellular and most of it consists of ECM, chondrocytes (the sole resident cell type in adult articular cartilage) are not only regulators of matrix homeostasis but are increasingly recognized as active contributors to the joint immune microenvironment. They produce cytokines, chemokines, and matrix-degrading enzymes, and respond to inflammatory mediators produced by resident and infiltrating immune cells in the joint, thereby amplifying inflammation-associated joint pathology, particularly in OA (21, 22). For this reason, defining how TIMP-3 influences gene expression in articular cartilage is also relevant to understanding its potential impact on immune-related pathways in the joint. As chondrocytes reside in an avascular, low-oxygen extracellular environment, where physiological oxygen tension fluctuates between approximately 1% and 6% (23, 24), we exposed cartilage cultures to TIMP-3 under both normoxic (21% O_2_) and hypoxic (3% O_2_) conditions.

## 2 Materials and methods

### 2.1 Isolation and culture of femoral head articular cartilage

**Figure 1:**
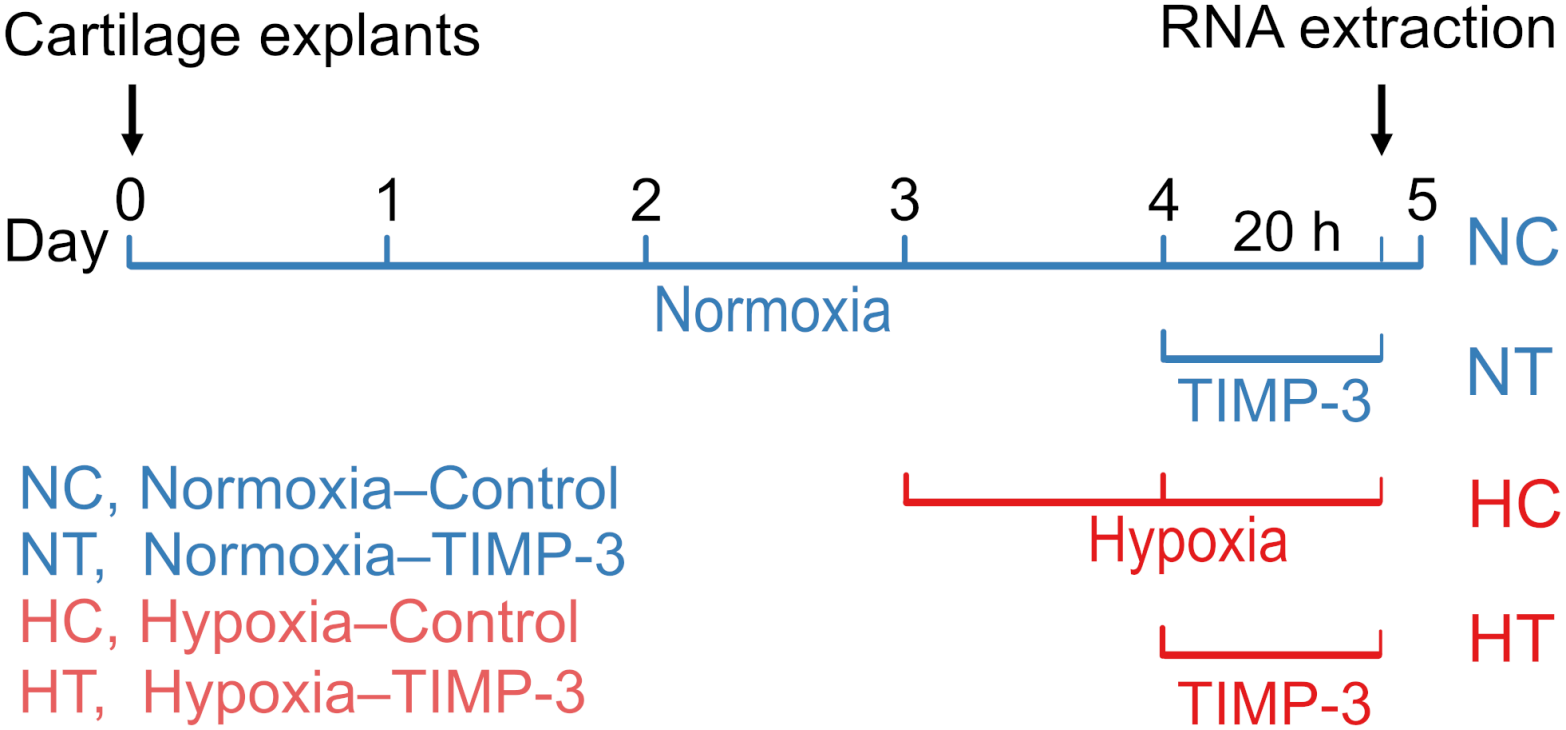
Experimental design. Femoral head articular cartilage samples from 16 mice were harvested in pairs and each pair was placed in one well of a 96-well plate with serum-free Opti-MEM supplemented with 1% penicillin-streptomycin, maintained under standard normoxic conditions (21% O_2_, 5% CO_2_, 37°C). After three days, half of the cultures were transferred to a hypoxia incubator (3% O_2_). Following 24 hours of normoxic or hypoxic incubation, cultures were treated with recombinant human TIMP-3 (100 nM; 2.6 µg/ml) or vehicle for 20 hours, and RNA was extracted for RNA sequencing (n = 4 per group). Group labels: NC, normoxia control; NT, normoxia TIMP-3; HC, hypoxia control; HT, hypoxia TIMP-3.

Articular cartilage was harvested from 4-week-old C57BL/6 mice (Charles River Laboratories, Margate, UK) euthanized by carbon dioxide (CO_2_) inhalation, in accordance with the UK Animals (Scientific Procedures) Act of 1986 and local institutional guidelines. Hip joints were disarticulated to expose the femoral heads. Cartilage caps were then avulsed using forceps, as previously described, and used as explants for *ex vivo* culture (19, 20). Mice were used at four weeks of age, when the femoral head cartilage caps consist predominantly of articular cartilage, as the secondary ossification centers are not fully mineralized yet (19, 20). Pairs of cartilage explants were placed in individual wells of a 96-well plate and cultured in serum-free Opti-MEM (Thermo Fisher Scientific, Waltham, MA, USA) supplemented with 1% penicillin-streptomycin (Thermo Fisher Scientific) under standard normoxic culture conditions (approximately 21% O_2_, 5% CO_2_, 37°C) for three days to recover from avulsion injury (20). After this resting period, cultures were either maintained under normoxia or transferred to a hypoxic incubator (3% O_2_, 5% CO_2_, 37°C). Hypoxia was modeled at 3% O_2_ to approximate physiological conditions in avascular articular cartilage, where oxygen tension typically ranges between 1% and 6% (23, 24). After 24 hours, cultures were treated with recombinant human TIMP-3 (R&D Systems, Abingdon, Oxon, UK) at 100 nM, (2.6 µg/ml) or vehicle control for 20 hours under normoxia or hypoxia. Each of the four experimental groups included four biological replicates (n = 16 in total). The experimental design is shown in **Figure 1**.

### 2.2 RNA extraction

Cartilage samples (two explants per sample) were transferred into 2 mL reinforced homogenization tubes containing 2.8 mm ceramic beads (Precellys lysing kit CK28R; Bertin Technologies, Montigny-le-Bretonneux, France) and 600 µl of QIAzol lysing reagent (QIAGEN, Venlo, The Netherlands). Samples were homogenized using a Precellys tissue homogenizer for four cycles of 40 seconds at 6500 rpm, with 20-seconds intervals on ice between each cycle.

Total RNA was extracted using the miRNeasy Micro kit (QIAGEN), incorporating on-column DNase treatment (QIAGEN) to remove genomic DNA. To optimize extraction from small samples, the manufacturer’s protocol was followed, with the wash buffer reconstituted using isopropanol instead of ethanol, as recommended. RNA concentration and purity were assessed using a NanoDrop One spectrophotometer (Thermo Fisher Scientific). RNA integrity was evaluated using the 2100 Bioanalyzer (Agilent Technologies, Santa Clara, CA, USA). The yield from individual cartilage samples (two explants each) ranged from 0.95 to 1.8 µg. All samples exhibited A260/A280 ratios > 1.9, A260/A230 ratios > 1.2 and RNA integrity numbers (RIN) between 5.4 and 8.2.

### 2.3 RNA sequencing

cDNA libraries were generated using the QuantSeq 3’ mRNA-Seq Library Prep kit FWD for Illumina (Lexogen, Vienna, Austria), starting with 250 ng of total RNA per sample. Library quality was assessed using a Bioanalyzer High Sensitivity DNA chip (Agilent Technologies), and DNA concentration was quantified using a Qubit fluorometer (Thermo Fisher Scientific). The average library size was 300 bp, with an average DNA concentration of 2.5 ng/µL, resulting in an average molarity of 12.5 nM (range: 7–17 nM). Libraries were pooled to a final concentration of 7 nM.

RNA sequencing (RNA-seq) was performed by Lexogen using a 75 bp single-end run on an Illumina NextSeq platform (Illumina, San Diego, CA, USA), generating 3–5 million reads per sample. The raw RNA-seq data (FASTQ files) have been deposited in BioStudies under ArrayExpress accession number E-MTAB-15388 and can be accessed at https://www.ebi.ac.uk/biostudies/studies?query=E-MTAB-15388.

### 2.4 Analysis of RNA-seq data

Sequence quality was assessed using FastQC (v0.11.9; https://www.bioinformatics.babraham.ac.uk/projects/fastqc/), and adapter sequences were trimmed using Cutadapt (v2.1; Python v3.6.6). Cleaned reads were aligned to the *Mus musculus* reference genome (GRCm39.107, Ensembl) using HISAT2 (v2.2.0; https://daehwankimlab.github.io/hisat2/). The resulting SAM files were converted to sorted BAM files using SAMtools. Raw transcript counts, obtained with FeatureCounts (v2.0.3), were imported into R (v4.2.2; http://www.r-project.org) and analyzed with the edgeR package (v3.38.5; Bioconductor v3.15). Lowly expressed genes were filtered out with the filterByExpr function, and counts per million (CPM) were calculated after normalization for library size. Differential expression was assessed using the quasi-likelihood F-test, based on a negative binomial model.

Heatmaps were generated with GENESIS v1.8.1 (25). Functional enrichment analysis was performed using the Database for Annotation, Visualization and Integrated Discovery (DAVID; DAVID Knowledgebase v2025_2; https://davidbioinformatics.nih.gov/), querying Gene Ontology Biological Process (GO:BP) terms and Kyoto Encyclopedia of Genes and Genomes (KEGG) pathways (26, 27). The background gene list consisted of all genes detected as expressed in our mouse articular cartilage samples after filtering lowly expressed genes with the filterByExpr function (edgeR) prior to differential expression analysis. Enriched categories were ranked based on the EASE score (a modified Fisher’s exact P-value implemented by DAVID) and the false discovery rate (FDR). Potential protein-protein interaction (PPI) networks were constructed with the Search Tool for the Retrieval of Interacting Genes/Proteins (STRING v12.0; https://string-db.org/), based on known and predicted interactions of proteins encoded by the genes of interest. Data visualization was carried out in R (v4.2.2) using the ggplot2 package (https://ggplot2.tidyverse.org/) and in GraphPad Prism (v8.0.2; GraphPad Software, San Diego, CA, USA). Venn diagrams were generated using the online tool developed by VIB/UGent (https://bioinformatics.psb.ugent.be/webtools/Venn/).

### 2.5 RT-qPCR validation assays

One hundred nanograms of total RNA were reverse transcribed in 20 µL reactions using the High-Capacity cDNA Reverse Transcription kit (Applied Biosystems, Thermo Fisher Scientific) following the manufacturer’s protocol. The resulting complementary DNA (cDNA) was diluted to 40 µL with RNase-free water and used as a template for quantitative real-time PCR (qPCR), performed using TaqMan gene expression assays (Applied Biosystems, Thermo Fisher Scientific) and Brilliant III qPCR master mix (Agilent Technologies). Negative controls included no-RT controls (RNA processed without reverse transcriptase) and no template controls. All reactions were run in duplicate on the AriaMx Real-Time PCR System (Agilent Technologies), with cycle times and temperatures as per the Brilliant III qPCR protocol (Agilent Technologies). Gene expression was analyzed using the comparative Cq (ΔΔCq) method according to Applied Biosystems guidelines. Expression levels were normalized to *Hprt* reference gene and reported as log_2_ fold change (FC) relative to the mean of the control group. TaqMan assays used included *Saa3* (Mm00441203_m1), *Il17b* (Mm01258783_m1), *Mmp3* (Mm00440295_m1), *Lcn2* (Mm01324470_m1), *Pbk* (Mm01313789_g1), *Racgap1* (Mm00488845_m1), *Hprt* (Mm00446968_m1).

### 2.6 Statistical analysis

RNA-seq data were analyzed for differential expression using edgeR (R v4.2.2; edgeR v3.38.5). A quasi-likelihood F-test was applied on raw counts, using a negative binomial generalized linear model. When comparing hypoxic with normoxic samples, transcripts with FDR < 0.05 and an absolute log_2_FC (|log_2_FC|) > 0.58 (equivalent to |FC| > 1.5) were considered differentially expressed. When comparing TIMP-3–treated and control samples under normoxic or hypoxic conditions, differentially expressed transcripts were identified based on P < 0.01 and |log_2_FC| > 0.58. Selected expression changes were validated by RT-qPCR with TaqMan gene expression assays, as described above. RT-qPCR log_2_FC (-ΔΔCq) values were analyzed using unpaired two-sided Welch’s t-tests for predefined pairwise comparisons (R v4.2.2).

## 3 Results

Articular cartilage explants from the femoral heads of mice were incubated in serum-free medium for 72 hours. Half of the cultures were then maintained in normoxia (approximately 21% O_2_), and half were transferred to hypoxic conditions (3% O_2_). After 24 hours, samples were either treated with vehicle or with 100 nM (2.6 µg/ml) TIMP-3 for 20 hours (experimental design in **Figure 1**). Total RNA was subsequently extracted, and RNA-seq was performed on control and TIMP-3–treated samples under both oxygen conditions.

### 3.1 Transcriptomic analysis shows a characteristic hypoxia response in mouse articular cartilage

Before evaluating the transcriptional response to TIMP-3 exposure, we analyzed the effect of hypoxia alone. Untreated control cultures were maintained under hypoxia for a total of 44 hours, corresponding to the combined duration of the 24-hour preincubation and the subsequent 20-hour TIMP-3 treatment. Differentially expressed transcripts between hypoxic and normoxic conditions were identified at FDR < 0.05 and |FC| > 1.5 (|log_2_FC| > 0.58). In total, 929 transcripts (corresponding to 920 annotated genes) were upregulated, and 784 transcripts (774 genes) were downregulated under hypoxia (summary in **Table 1** and complete lists in **Supplementary Files 1, 2**).

**Table 1:** Transcriptomic changes in cartilage in response to hypoxia.

Effect of hypoxia	Transcripts	Genes
Up	929	920
Down	784	774
Unaffected	10,798	10,646

Articular cartilage explants from mouse femoral heads were cultured under normoxic conditions for three days. Cultures were then either maintained in normoxia or transferred to hypoxic conditions (3% O_2_). After 44 hours, RNA-seq was performed. Differentially expressed genes were identified by comparing hypoxic and normoxic samples (n = 4), using the edgeR package. Thresholds: FDR < 0.05 and |log_2_FC| > 0.58 (equivalent to |FC| > 1.5). Gene lists are in **Supplementary Files 1, 2**. FC, fold change.

**Figure 2:**
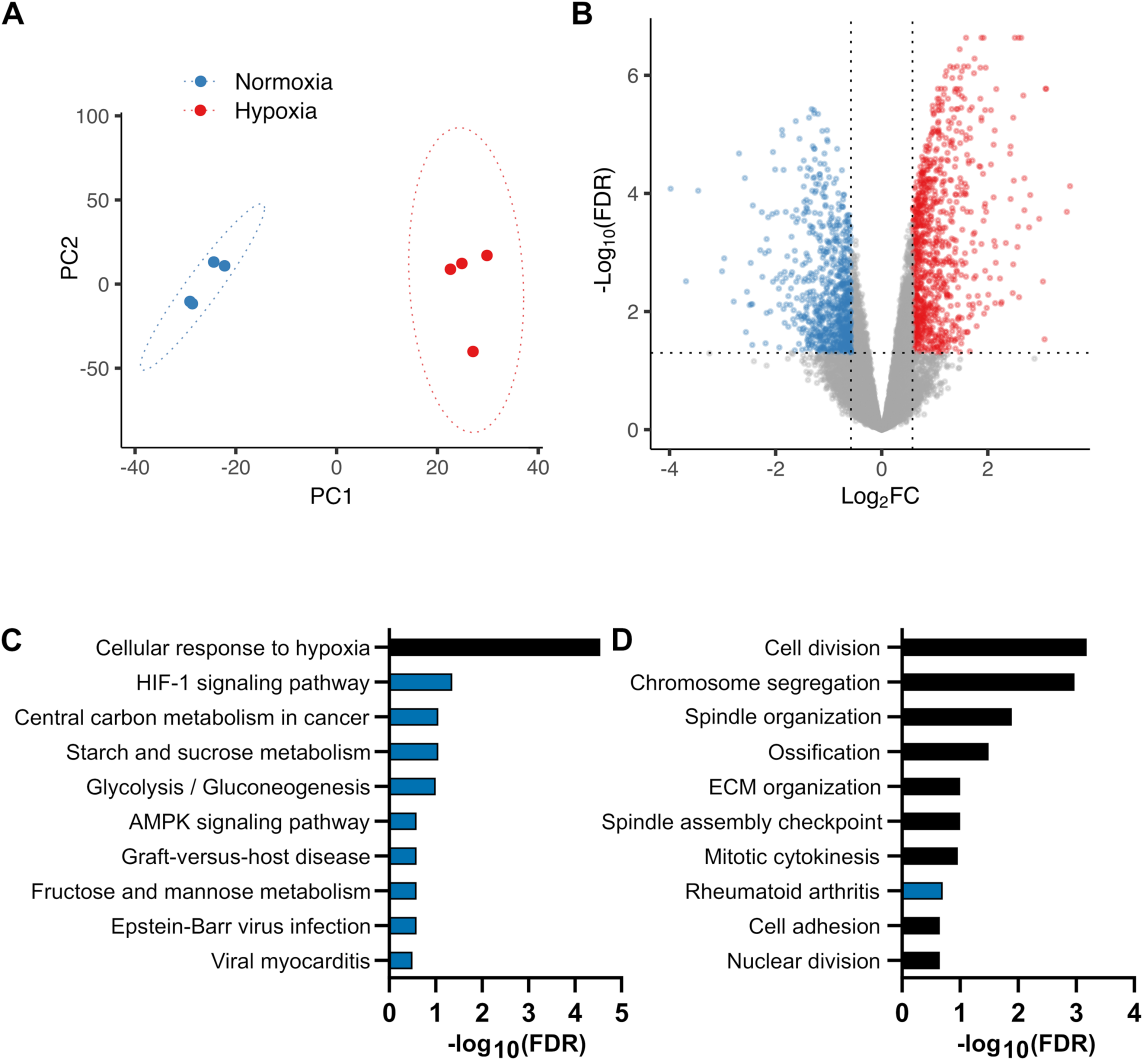
RNA-seq analysis of cartilage in response to hypoxia (3% O_2_, 44 hours) versus normoxia (21% O_2_). Articular cartilage explants were cultured and processed for RNA-seq as described in the legend to **Table 1**. **(A)** Principal component analysis (PCA) plot showing sample clustering (n = 4 per group). Ellipses are shown for visualization only. **(B)** Volcano plot showing differentially expressed transcripts. Grey: no change, red: upregulated, blue: downregulated. Thresholds: FDR < 0.05, |log_2_FC)| > 0.58 (|FC| > 1.5). Gene lists are in **Supplementary Files 1, 2**. **(C, D)** DAVID functional enrichment analysis of the 920 hypoxia-upregulated **(C)** and 774 hypoxia-downregulated **(D)** genes. The top 10 enriched Gene Ontology Biological Process (GO:BP) terms (black) or KEGG pathways (blue), ranked by FDR, are shown. Corresponding gene lists are in **Supplementary File 3**. FC, fold change; FDR, false discovery rate; HIF-1, hypoxia inducible factor 1; AMPK, adenosine monophosphate-activated kinase; ECM, extracellular matrix.

The magnitude of changes in gene expression was not limited to modest fold differences, as 40% of differentially expressed transcripts showed changes greater than 2-fold, and 4% greater than 4-fold (**Supplementary Files 1, 2**). **Figures 2A, B** provide an overview of the RNA-seq analysis, with the principal component analysis (PCA) showing sample clustering (**A**) and the volcano plot showing differential gene expression (**B**).

Using a conservative expressed-gene background, DAVID enrichment analysis revealed overrepresentation of upregulated GO:BP terms and KEGG pathways including “Cellular response to hypoxia”, “HIF-1 signaling” and “Glycolysis”. Downregulated genes were enriched for pathways associated with mitosis, indicating reduced cell proliferation. Together, these findings confirm a characteristic transcriptional response to hypoxia. Additional downregulated GO:BP terms included “Extracellular matrix organization”, with cartilage degrading enzymes such as *Adamts4*, *Adamts5* and several *Mmps*, as well as “Ossification”, including genes such as Runt-related transcription factor 2 (*Runx2)* and *Adamts12*, consistent with a cartilage-specific hypoxia response (**Figures 2C, D**). The corresponding gene lists for each enriched category are in **Supplementary File 3**.

### 3.2 Genes differentially expressed in response to TIMP-3

The transcriptional effects of TIMP-3 under normoxia and hypoxia were evaluated by differential expression analysis. At FDR < 0.05, TIMP-3 did not alter the expression of any genes under either oxygen condition. However, when a less stringent threshold was applied (unadjusted P < 0.01 combined with |FC| > 1.5, corresponding to |log_2_FC| > 0.58), several transcripts were differentially expressed. This approach was used to identify potential false negatives resulting from multiple testing correction (28), with the caveat that only gene expression changes subsequently validated by RT-qPCR can be considered true positives. Under normoxic conditions, 26 transcripts were upregulated and 29 downregulated, all with annotated gene symbols. Under hypoxia, 19 transcripts were upregulated (corresponding to 18 genes; one transcript lacking a gene symbol [ENSMUSG00002076161] was excluded from further analysis) and 10 transcripts were downregulated, all with gene symbols (summary in **Table 2**). The complete lists of TIMP-3–responsive genes under normoxia and hypoxia are in **Supplementary Files 4, 5**.

**Table 2:** Gene expression changes in cartilage in response to TIMP-3.

Change	TIMP-3 vs Ctrl FDR < 0.05	TIMP-3 vs Ctrl P < 0.01, |log_2_FC| > 0.58
Normoxia		
Up	0	26
Down	0	29
Unaffected	12,514	12,459
Hypoxia		
Up	0	18
Down	0	10
Unaffected	12,081	12,053

Articular cartilage explants from mouse femoral heads were cultured under normoxic conditions for three days. Cultures were then either maintained in normoxia or transferred to hypoxic conditions (3% O_2_). After 24 hours, samples were treated with recombinant TIMP-3 (100 nm; 2.6 µg/ml) or vehicle for 20 hours, and RNA-seq was performed. Differentially expressed genes were identified by comparing TIMP-3-treated samples with their respective normoxic or hypoxic controls (n = 4), using the edgeR package. Gene lists are in **Supplementary Files 4, 5**. Ctrl, control; FC, fold change; |log_2_FC| > 0.58 is equivalent to |FC| > 1.5.

### 3.3 TIMP-3 upregulates *Saa3* under both normoxia and hypoxia

**Figure 3:**
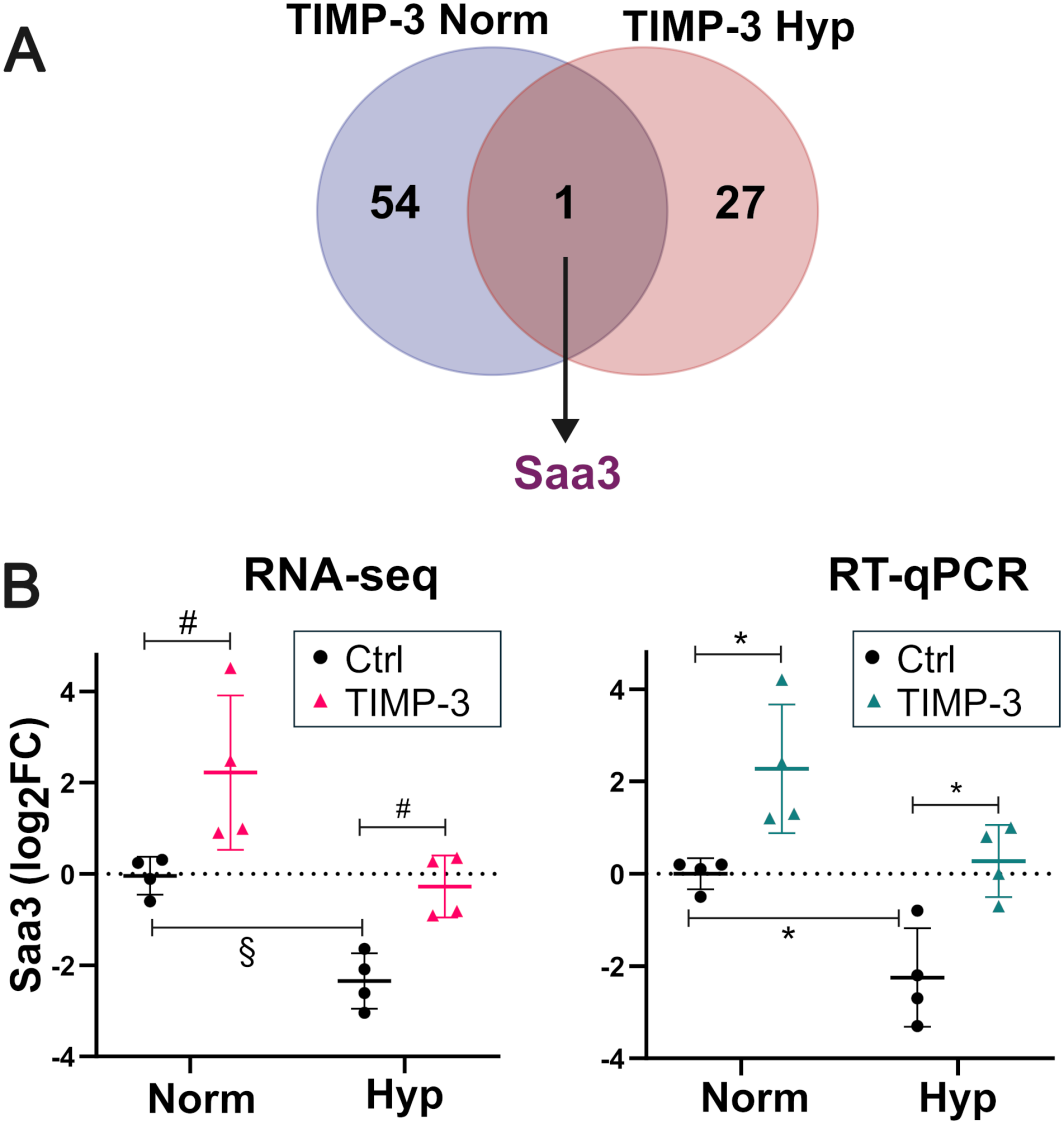
TIMP-3 upregulates *Saa3* gene expression in cartilage under normoxia and hypoxia. Articular cartilage explants were cultured and processed for RNA-seq as described in the legend to **Table 2**. **(A)** Venn diagram showing overlap of genes differentially regulated in response to TIMP-3 under normoxia (Norm; 21% O_2_) and hypoxia (Hyp; 3% O_2_). **(B)** RT-qPCR validation of *Saa3*. RNA-seq (left) and RT-qPCR (right) data shown as log_2_FC versus the mean of the controls (mean ± SD, n = 4). RNA-seq: log_2_FC were calculated from normalized CPM values; § FDR < 0.05 (hypoxia effect), # P < 0.01 (TIMP-3 effect) by quasi-likelihood F-test applied to raw counts (edgeR). RT-qPCR: *P < 0.05 by unpaired two-sided Welch’s t-test on log_2_FC values (-ΔΔCq). Ctrl, control; FC, fold change.

Following the identification of genes differentially regulated by TIMP-3 based on unadjusted P-values, a subset was selected for RT-qPCR validation. We focused on TIMP-3–responsive genes under both normoxia and hypoxia. Serum amyloid A3 *(Saa3)* was the only gene differentially expressed in both conditions (**Figure 3A**). RT-qPCR confirmed its induction by TIMP-3 in normoxia and hypoxia, with fold changes relative to untreated controls that mirrored the RNA-seq data (**Figure 3B**). Despite similar TIMP-3–mediated fold induction of *Saa3* in both oxygen conditions, hypoxia alone downregulated *Saa3*, resulting in lower absolute expression levels following TIMP-3 treatment under hypoxia than under normoxia (normalized CPM in TIMP-3–treated samples, median [interquartile range (IQR)]: normoxia, 42 [22–109]; hypoxia, 11 [7–15]; FDR = 0.025 by quasi-likelihood F-test in edgeR applied to raw counts).

### 3.4 TIMP-3 upregulates IL-17 pathway genes in normoxic conditions

**Figure 4:**
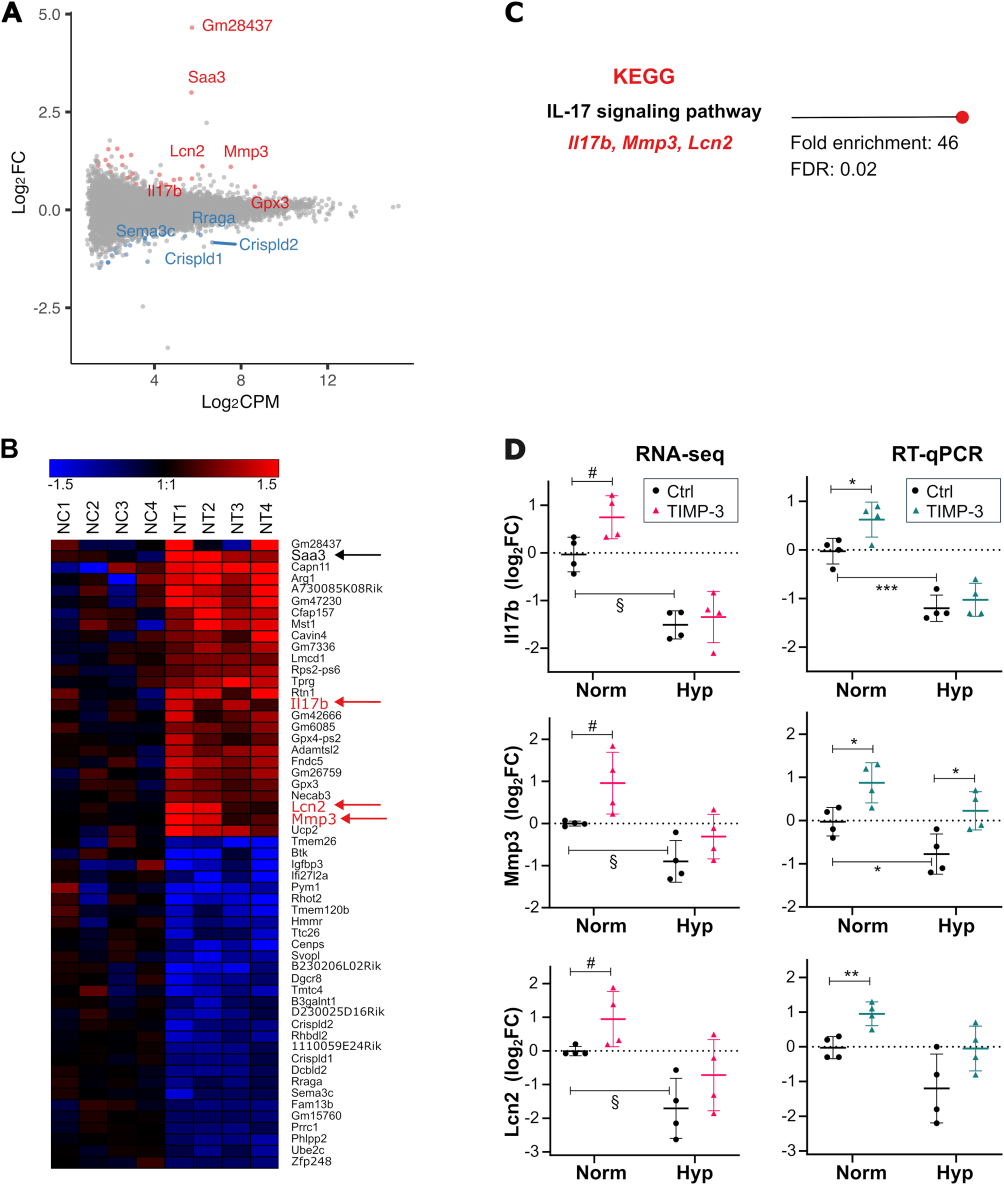
RNA-seq analysis of TIMP-3–treated cartilage under normoxia. **(A)** MA plot showing differential expression between TIMP-3–treated and control samples, with log_2_FC plotted against average log_2_CPM expression (n = 4). Grey: no change, red: upregulated, blue: downregulated transcripts (P < 0.01 by quasi-likelihood F-test in edgeR, |log_2_FC| > 0.58). The 10 most highly expressed regulated genes are labelled. Complete gene lists are in **Supplementary File 4**. **(B)** Heatmap of differentially expressed genes (log_2_FC versus the mean of the controls). Genes are ordered by hierarchical clustering. Sample labels: NC1-4: normoxia controls; NT1-4: normoxia TIMP-3–treated. **(C)** Sole significantly enriched pathway (FDR < 0.05) from DAVID analysis of 26 upregulated genes. As only one KEGG pathway passed the significance threshold, it is shown individually together with its enrichment score (Fold enrichment) and FDR for visual consistency within the multipanel figure. **(D)** RT-qPCR validation. RNA-seq (left) and RT-qPCR (right) data shown as log_2_FC versus the mean of the controls (mean ± SD, n = 4). RNA-seq: log_2_FC were calculated from normalized CPM values; § FDR < 0.05 (hypoxia effect), # P < 0.01 (TIMP-3 effect) by quasi-likelihood F-test applied to raw counts (edgeR). RT-qPCR: *P < 0.05, **P < 0.01, ***P < 0.001 by unpaired two-sided Welch’s t-test on log_2_FC values (-ΔΔCq). CPM, counts per million; Ctrl, control; FC, fold change; Norm, normoxia; Hyp, hypoxia.

As the overlap between TIMP-3-responsive genes in normoxia and hypoxia was minimal, we next examined whether TIMP-3 affected gene expression differently under the two oxygen conditions, focusing first on normoxia. TIMP-3–induced gene expression changes were visualized with an MA plot (**Figure 4A**), showing log_2_ expression ratios (M; log_2_FC) versus average log_2_ expression (A; log_2_CPM), and a heatmap of the four biological replicates (**Figure 4B**). Finally, DAVID functional enrichment analysis was conducted to identify overrepresented GO:BP terms and KEGG pathways (**Figure 4C**).

At an FDR < 0.05, no GO:BP terms were enriched among either the upregulated or downregulated genes, and no KEGG pathways were overrepresented among the downregulated genes. However, the KEGG IL-17 signaling pathway was significantly enriched among TIMP-3–upregulated genes (**Figure 4C**). Interleukin17b *(IL17b)*, matrix metalloproteinase 3 *(Mmp3)* and lipocalin 2 *(Lcn2)*, the three genes associated with this pathway, ranked among the top ten differentially expressed genes by expression level (CPM; **Figure 4A** and **Supplementary File 4**) and were selected for validation.

RT-qPCR confirmed their significant induction by TIMP-3 under normoxia (**Figure 4D**). All three genes were downregulated by hypoxia alone, an effect also confirmed by RT-qPCR. These results were consistent with the RNA-seq data in both direction and magnitude of change (**Figure 4D**). In all cases, RT-qPCR P-values for differentially expressed genes were < 0.05, except for *Lcn2*, whose hypoxia-induced downregulation mirrored RNA-seq results (mean log_2_FC = -1.2 by RT-qPCR; -1.4 by RNA-seq) but with a P-value of 0.087.

Interestingly, RT-qPCR revealed a significant *Mmp3* induction by TIMP-3 not only under normoxia (as detected by RNA-seq and confirmed by RT-qPCR; **Figure 4D**), but also under hypoxia, with a mean log_2_FC of 0.96 (P = 0.029). This supports the trend observed by RNA-seq, which showed a log_2_FC of 0.6 that did not reach statistical significance (P = 0.051; **Figure 4D**). However, as with *Saa3*, hypoxia downregulated *Mmp3*, so that absolute expression levels upon TIMP-3 treatment remained lower under hypoxia than under normoxia (normalized CPM in TIMP-3–treated samples, median [IQR]: normoxia, 216 [153–303]; hypoxia, 97 [77–122]; FDR = 0.037 by quasi-likelihood F-test in edgeR applied to raw counts).

### 3.5 TIMP-3 downregulates cell proliferation-associated genes under hypoxia

**Figure 5:**
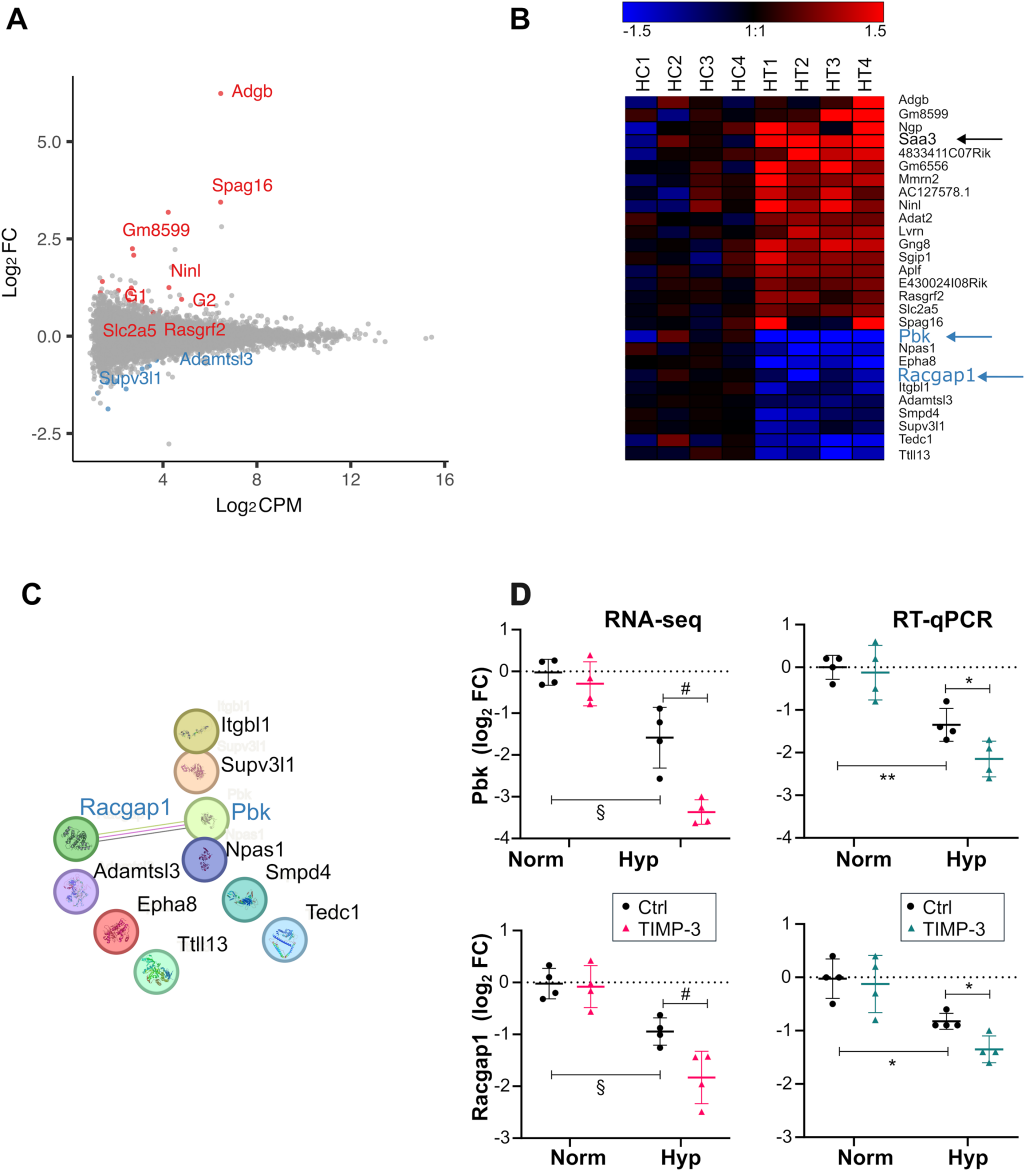
RNA-seq analysis of TIMP-3–treated cartilage under hypoxia. **(A)** MA plot showing differential expression between TIMP-3–treated and control articular cartilage, with log_2_FC plotted against average log_2_CPM expression (n = 4). Grey: no change, red: upregulated, blue: downregulated (P < 0.01, |log_2_FC| > 0.58). The 10 most highly expressed regulated genes are labelled. G1 and G2 correspond to E430024I08Rik and AC127578.1 respectively. Complete gene lists are in **Supplementary File 5**. **(B)** Heatmap of differentially expressed genes (log_2_FC versus the mean of the controls). Genes are ordered by hierarchical clustering. Sample labels: HC1-4: hypoxia controls; HT1-4: hypoxia TIMP-3-treated. **(C)** Protein–protein interaction (PPI) network corresponding to genes downregulated by TIMP-3 under hypoxia (P <0.01 by quasi-likelihood F-test in edgeR, |log_2_FC| > 0.58), generated using STRING (default interaction score ≥ 0.400). All nodes represent the initially filtered gene list and are included to show the network context and highlight that only *Pbk* and *Racgap1* display a documented interaction. Line colors indicate evidence type: green, text mining; pink, experimental; black, co-expression. Combined interaction score: 0.711. **(D)** RT-qPCR validation. RNA-seq (left) and RT-qPCR (right) data shown as log_2_FC versus the mean of the controls (mean ± SD, n = 4). RNA-seq: log_2_FC were calculated from normalized CPM values; § FDR < 0.05 (hypoxia effect), # P < 0.01 (TIMP-3 effect) by quasi-likelihood F-test applied to raw counts (edgeR); RT-qPCR: *P < 0.05, **P < 0.01 by unpaired two-sided Welch’s t-test on log_2_FC values (-ΔΔCq). CPM, counts per million; Ctrl, control.

We performed similar analyses under hypoxia. TIMP-3–induced gene expression changes were visualized with an MA plot, also showing the average log_2_ expression (**Figure 5A**), and a heatmap of the four replicates (**Figure 5B**). Finally, DAVID functional enrichment analysis was conducted to identify overrepresented GO:BP terms and KEGG pathways among the upregulated or downregulated genes. No significantly enriched terms or pathways were identified at an FDR < 0.05 in either gene set. As a follow-up, STRING analysis was conducted, revealing a potential link between PDZ-binding kinase (*Pbk)*, also known as T-LAK cell-originated protein kinase *(Topk*), the most strongly downregulated gene (**Figure 5B** and **Supplementary File 5**), and Rac GTPase-activating protein 1 (*Racgap1*; **Figure 5C**). No protein-protein interactions were detected among the TIMP-3–upregulated genes.

*Pbk* and *Racgap1* were selected for validation, and their changes in expression were confirmed by RT-qPCR (**Figure 5D**). Both genes were downregulated by hypoxia alone and further downregulated by TIMP-3.

## 4 Discussion

In this study, we examined the transcriptional response of mouse articular cartilage explants to recombinant TIMP-3 under normoxia and hypoxia. Given that articular chondrocytes normally reside in a low-oxygen environment (1-6% oxygen), hypoxia was modeled at 3% oxygen to approximate physiological conditions (23, 24). As reproducing the *in vivo* environment is challenging, normoxia was included for comparison and to provide a broader context for interpreting the transcriptional effects of exogenous TIMP-3 on cartilage.

Mammalian articular cartilage explants, including murine explants, have been widely used to investigate mechanisms underlying cartilage degradation observed *in vivo* in experimental OA (20, 29, 30). Genes regulated in whole joints following surgical destabilization are similarly regulated in articular cartilage *in vivo* (30), and similar gene expression changes can be reproduced in cartilage explants after explantation (avulsion) injury (20). Importantly, after a resting period allowing injury-induced responses to return to control level, explants remain responsive to biologically relevant stimuli that drive gene regulation *in vivo*. Notably, using the same mouse femoral head cartilage explant model employed here, Chong et al. showed significant gene expression responses to fibroblast growth factor 2 (FGF-2), a key mediator of injury-induced gene regulation in destabilized joints (20). These findings support the suitability of this *ex vivo* cartilage explant system for studying gene expression responses relevant to the whole joint *in vivo.*

Explants were rested for 72 hours to allow recovery from avulsion injury, as most injury-induced genes return to baseline within 48 hours (20, 29). After this recovery period, exposure to hypoxia elicited a robust transcriptional response, with upregulation of canonical hypoxia-inducible factor (HIF) targets such as nitric oxide synthase 2 (*Nos2)* and vascular endothelial growth factor a (*Vegfa)* (31, 32), and glycolytic genes mediating the metabolic shift under low oxygen (33), consistent with previous reports (34, 35) and thus validating the hypoxia model used. In parallel, matrix-degrading enzymes (*Adamts4*, *Adamts5* and several *Mmps*) and genes associated with hypertrophy and mineralization, such as *Runx2* and alkaline phosphatase (*Alpl)*, were downregulated, in agreement with earlier findings (36–38). In contrast, the ECM component collagen type II alpha 1 *(Col2a1)*, typically upregulated by hypoxia in cartilage (34, 36, 37), remained unchanged. This aligns with Thoms et al., who reported that short-term hypoxia (72 hours) did not induce *Col2a1* in cartilage from 6-week-old mice, unlike in human and porcine cartilage (39). Similarly, the hypoxia-responsive SRY-box transcription factor 9 (*Sox9)*, a key driver of *Col2a1* expression, was not affected, again consistent with Thoms et al. (39). However, long-term hypoxia (7 days) in mouse tibial explants increased *Col2a1* and *Sox9* expression (38), suggesting that mouse cartilage can regulate these genes under hypoxia, but their response may be slower or less sensitive than in other species. Collectively, these results confirmed that our explants responded to hypoxia in ways consistent with prior studies.

Despite a strong transcriptional response to hypoxia, recombinant TIMP-3 had limited effects on gene expression under either normoxic or hypoxic conditions, in contrast to the marked transcriptional changes reported by Xia et al. in cardiomyocytes (18), possibly due to differences in experimental model and conditions. The limited transcriptional effect of TIMP-3 in cartilage likely reflects its predominant mode of action at the protein level, primarily through direct inhibition of ECM–degrading enzymes (3, 4, 6, 9). However, when less conservative statistical thresholds were applied, a small subset of genes showed modest changes, which were nonetheless confirmed by RT-qPCR. These results support the notion that stringent multiple-comparison corrections, while reducing false positives, can also mask subtle yet reproducible effects (28), whose potential biological relevance warrants consideration.

TIMP-3 increased the expression of several inflammation-associated genes. The acute-phase gene *Saa3* was upregulated under both normoxia and hypoxia, whereas genes belonging to the IL-17 signaling pathway (*Il17b*, *Lcn2* and *Mmp3*) were increased mainly under normoxia. All these genes, described as HIF-responsive and inflammation-associated in other tissues (40–42), were instead downregulated by hypoxia alone in our cartilage explants. This observation suggests that chondrocytes, which reside in a chronically hypoxic environment, may have adapted to limit an inflammation-associated component of the HIF transcriptional response, consistent with the role of hypoxia in maintaining cartilage homeostasis (36, 37). *Saa3* remained significantly induced by TIMP-3 under both normoxic and hypoxic conditions, indicating that TIMP-3 can at least partially override hypoxia-associated suppression for selected targets and suggesting that *Saa3* may be particularly sensitive to TIMP-3–dependent regulation, consistent with a stronger transcriptional response than the other genes identified. However, because hypoxia lowers baseline expression, absolute *Saa3* levels remained lower under hypoxia than normoxia despite similar fold induction. These results suggest that TIMP-3 may promote gene expression changes with potential pro-inflammatory and catabolic consequences that are more likely to have a greater biological impact under more oxygenated conditions.

Serum amyloid A (SAA) acute-phase proteins are induced by inflammatory stimuli and are widely used as inflammatory markers (43). In mice, *Saa3* is the predominant extrahepatic isoform, while in humans *SAA3* is a pseudogene, but *SAA1* and *SAA2* are homologous to mouse *Saa3* and are also expressed extrahepatically (44). *Saa3*, or human *SAA1/SAA2*, are expressed in RA and OA joints by multiple cell types, including chondrocytes, where they amplify inflammatory and catabolic responses by enhancing *Mmp* expression (45, 46). Recent studies also support a role for endogenous SAA in bone loss associated with infection in humans and chronic parathyroid hormone administration in mice (47, 48). Similarly, the IL-17 family, particularly IL-17A, is pro-inflammatory and implicated in autoimmune disease and OA (49). Although less studied, IL-17B is highly expressed in cartilage and in RA and OA synovium and generally promotes inflammation (50, 51); its neutralization in experimental arthritis suppresses bone destruction (52). MMP3 and LCN2 are downstream targets of IL-17 and are also induced by other inflammatory stimuli (53, 54). MMP3 is a well-established mediator of cartilage pathology, including in OA (55), while LCN2, a multifunctional protein expressed in various tissues (56), is also expressed in chondrocytes and osteoblasts in OA joints, and contributes to cartilage degradation (57–59). In transgenic mice, LCN2 bone-specific overexpression inhibits bone formation and increases resorption, resulting in decreased bone mass (60). Together, these observations indicate that TIMP-3–upregulated genes participate in pro-inflammatory and catabolic pathways affecting cartilage and bone.

This finding is unexpected, as TIMP-3 generally functions to preserve articular cartilage homeostasis by inhibiting catabolic MMP and ADAM/ADAMTS enzymes (6, 9, 10). However, cartilage-specific overexpression of TIMP-3 in transgenic mice has been shown to impair bone mass and structure (16). In this model, chondrocytes highly expressing TIMP-3 are likely incorporated into the cartilage template during endochondral ossification, and many hypertrophic chondrocytes differentiate into osteoblasts and osteocytes (61, 62), potentially transferring TIMP-3 into bone. It is therefore plausible that cartilage-specific genes may contribute to bone phenotypes. Scilabra et al. (7) recently proposed that, in TIMP-3 transgenic mice, persistent inhibition of the sheddase ADAM10, observed *in vitro* following TIMP-3 overexpression, may reduce cleavage and signaling of the Notch receptor (63), causing dysregulated bone development and metabolism. Our data suggest an additional, non-enzymatic mechanism by which TIMP-3 could adversely affect bone, involving induction of gene expression programs associated with potential pro-inflammatory and catabolic effects. Because endochondral ossification occurs in a vascularized (and thus oxygenated) environment (38), this hypothesis is supported by the observed TIMP-3–mediated induction of inflammation-associated genes primarily under normoxia.

Mechanistically, TIMP-3 has multiple binding partners and may therefore affect gene expression through several pathways. It interacts with the LRP-1 receptor (8, 9), which mediates its endocytosis and the clearance of TIMP-3–bound MMPs. Although signaling downstream of this binding has not been described, LRP-1 engagement by diverse ligands can activate extracellular-signal regulated kinase (ERK) and other signaling cascades (64–67). ERK activation in turn can induce *Mmp3* expression in fibroblasts and chondrocytes (68, 69), and *Lcn2* expression in other cell types (70, 71). TIMP-3 also binds glycosaminoglycan chains of sulfated proteoglycans in the ECM, including heparan sulphate (HSPGs) (10, 11), which stabilizes it extracellularly but has not been directly linked to signaling. In other contexts, binding of heparin-binding angiogenic factors (CCN family members, including CYR61/CCN1 and CTGF/CCN2) to HSPGs and β1 integrins (such as α6β1) can trigger ERK activation and increase *Mmp3* expression (72). While TIMP-1 and TIMP-2 have been reported to interact with β1 integrins (73, 74), to the best of our knowledge TIMP-3’s only documented integrin interaction is with the intracellular C-terminal region of α7 (ITGA7) (75). Together, these observations suggest that TIMP-3 might regulate gene expression via LRP-1–mediated signaling. Although there is no direct evidence, it is also conceivable that TIMP-3 could act through an HSPG-integrin mechanism, distinct from the described α7 C-terminal interaction.

TIMP-3 also downregulated two cell-cycle dependent genes, *Pbk* and *Racgap1*, which were inhibited by hypoxia alone and further decreased upon TIMP-3 treatment. Both genes are highly expressed in proliferating cells and are well characterized in cancer, where their expression correlates with increased invasiveness and poor prognosis (76–78). Specifically, PBK/TOPK is a mitotic kinase similar to mitogen-activated protein kinase kinase (MAPKK) that phosphorylates p38 and ERK, among other substrates (77), whereas RACGAP1 promotes cytokinesis (78). To the best of our knowledge, their potential function in cartilage is unknown, although *Pbk* is increased in OA cartilage and synovium (79, 80), and *Racgap1* has been detected in mouse chondrocytes treated with IL-1β (81). The observation that hypoxia, a cartilage protective environment that inhibits its mineralization (36, 37), downregulates *Pbk* and *Racgap1* suggests that their decreased expression is associated with maintenance of cartilage homeostasis. TIMP-3 further reduces their levels, potentially reinforcing this protective effect by limiting excessive chondrocyte proliferation.

When interpreting the results of this study, several limitations should be considered. Gene expression in cartilage explants was assessed at a single time point and using a single dose of TIMP-3. The selected dose reflects current knowledge of TIMP-3 biology and the available literature. Exogenous TIMP-3 treatment is challenging because the protein binds strongly to the ECM (10, 11) and is rapidly internalized via LRP-1 (8, 9), which is why most prior studies have relied on overexpression systems (7, 82). The TIMP-3 levels required to inhibit aggrecanases *in vivo* at the joint are not known, nor are the tissue concentrations achieved in transgenic overexpressing models, where the presence of the protein has been assessed mainly by indirect methods (16). Our chosen concentration was therefore guided by the few available studies using exogenous recombinant TIMP-3 that reported biological effects at microgram-range doses (12, 82, 83), including one study showing concordant effects between overexpression and exogenous treatment at these concentrations (82). The 20-hour time point was chosen based on a previous study in cartilage explants that examined gene expression at 4 and 20 hours (29). We acknowledge that dose response and multiple time point analyses will be required to better define the kinetics and specificity of TIMP-3–induced gene expression changes, which will be explored in future studies.

It should also be considered that gene expression changes were measured in an *ex vivo* cartilage explant model. Although this system reproduces gene expression responses observed in whole joints in experimental OA and is appropriate for exploratory mechanistic studies (20, 29, 30), it does not capture the full synovial, subchondral bone, immune and systemic context of the joint. Confirmation *in vivo* at the joint level will therefore be required during further therapeutic development of TIMP-3.

Finally, this study was designed to test whether TIMP-3, beyond its established MMP-inhibitory activity at the protein level, also affects gene expression, and transcriptomic analysis was therefore key to addressing this question. As this was an exploratory RNA-seq study with a limited sample size and therefore reduced statistical power, candidate genes were identified using a threshold based on unadjusted P-values and FC, followed by mandatory RT-qPCR validation. Only genes meeting these predefined selection criteria and independently validated by RT-qPCR were interpreted biologically, whereas genes from the preliminary filtered lists should be considered hypothesis-generating and will require independent validation before further interpretation. Determining whether these transcriptional changes translate into protein production at functionally relevant levels is essential, but represents a distinct objective that will require dedicated follow-up studies, validation across multiple experimental models, and increased replicate numbers. Thus, the mechanistic pathways suggested here remain to be directly verified.

Overall, our study provides the first evidence that TIMP-3 elicits transcriptional changes in cartilage, including modulation of genes associated with inflammatory and catabolic processes. The distinct responses observed under normoxic and hypoxic conditions emphasize the importance of accounting for oxygen tension in cartilage studies. While hypoxia triggered a strong transcriptional response, TIMP-3 influenced only a small set of genes in a context-dependent manner. These findings identify a previously unrecognized effect of TIMP-3 in cartilage that may help explain its off-target effects *in vivo* and should be considered in its therapeutic development for arthritic disease. Future studies should validate these transcriptional effects *in vivo*, clarify the underlying regulatory mechanisms, and determine whether they translate into protein-level and functional changes relevant to joint biology and TIMP-3 therapeutic development.

## Data Availability

The datasets presented in this study can be found in online repositories. The names of the repository/repositories and accession number(s) can be found in the article/**Supplementary Material**.
