# Supplementary material for: Exploratory transcriptomic analysis of mouse articular cartilage in response to tissue inhibitor of metalloproteinase 3 identifies inflammation-associated gene expression changes
Source: Front Immunol. 2026 Mar 11;17:1794078. doi: 10.3389/fimmu.2026.1794078 (PMC13027204; doi:10.3389/fimmu.2026.1794078)
Supplement: Supplementary file 1 [file Table1.docx]

Supplementary Material

Supplementary File Legends

**Supplementary File 1.** Transcripts upregulated in cartilage in response to hypoxia (FDR < 0.05, log_2_FC > 0.58; see **Table 1** legend). Hyp, hypoxia; Norm, normoxia; CPM, counts per million; FC, fold change; FDR, false discovery rate.

**Supplementary File 2.** Transcripts downregulated in cartilage in response to hypoxia (FDR < 0.05, log_2_FC < -0.58; see **Table 1** legend). Hyp, hypoxia; Norm, normoxia; CPM, counts per million; FC, fold change; FDR, false discovery rate.

**Supplementary File 3.** Gene lists for the top 10 enriched GO:BP terms and KEGG pathways among genes up- or downregulated in cartilage in response to hypoxia, identified by DAVID functional enrichment (see **Figure 2** legend). Column GeneRegulation indicates whether the genes contributing to each DAVID term were upregulated or downregulated in RNA-seq. EASE score, modified Fisher's exact P-value implemented by DAVID; FDR, false discovery rate.

**Supplementary File 4.** Genes differentially expressed in cartilage in response to TIMP-3 under normoxia (P < 0.01, |log_2_FC| > 0.58; see **Figure 4** legend). P-values were calculated using the quasi-likelihood F-test in edgeR. FDR-adjusted P-values were uniformly high (0.8-0.9) and are not reported; only genes subsequently validated by RT-qPCR were considered true positives. CPM, counts per million; FC, fold change.

**Supplementary File 5.** Genes differentially expressed in cartilage in response to TIMP-3 under hypoxia (P < 0.01, |log_2_FC| > 0.58; see **Figure 5** legend). P-values were calculated using the quasi-likelihood F-test in edgeR. FDR-adjusted P-values were uniformly high (0.8-0.9) and are not reported; only genes subsequently validated by RT-qPCR were considered true positives. CPM, counts per million; FC, fold change.
